# Neurometabolic profiles of autism spectrum disorder patients with genetic variants in specific neurotransmission and synaptic genes

**DOI:** 10.1038/s41598-025-20090-x

**Published:** 2025-10-17

**Authors:** Joana Vilela, Andreia C. Pereira, Inês R. Violante, Susana Mouga, Célia Rasga, João Xavier Santos, Hugo Martiniano, Ana Rita Marques, Guiomar Oliveira, Miguel Castelo-Branco, Astrid Moura Vicente

**Affiliations:** 1 Departamento de Promoção da Saúde e Prevenção de Doenças Não Transmissíveis, Instituto Nacional de Saúde Doutor Ricardo Jorge, Lisbon, Portugal; 2 Faculty of Sciences, BioISI-Biosystems & Integrative Sciences Institute, University of Lisboa, Lisbon, Portugal; 3 Coimbra Institute for Biomedical Imaging and Translational Research (CIBIT), Pólo das Ciências da Saúde, University of Coimbra, Azinhaga de Santa Comba, 3000-548 Coimbra, Portugal; 4 School of Biomedical Engineering and Imaging Sciences, Faculty of Life Sciences and Medicine, King’s College London, London, UK; 5 Institute of Nuclear Sciences Applied to Health (ICNAS), Pólo das Ciências da Saúde, University of Coimbra, Azinhaga de Santa Comba, 3000-548 Coimbra, Portugal; 6 Faculty of Medicine, Pólo das Ciências da Saúde, University of Coimbra, Azinhaga de Santa Comba, 3000-548 Coimbra, Portugal; 7 Centro de Desenvolvimento da Criança, Unidade de Neurodesenvolvimento e Autismo, Hospital Pediátrico, Centro Hospitalar e Universitário de Coimbra, Coimbra, Portugal; 8 Faculty of Medicine, University Clinic of Pediatrics, University of Coimbra, Coimbra, Portugal

**Keywords:** Autism spectrum disorder, Magnetic resonance spectroscopy, Glutamate, GABA, Synapse, Neurogenetics, Autism spectrum disorders, Synaptic transmission

## Abstract

Autism Spectrum Disorder (ASD) is a neurodevelopmental condition characterized by impaired social interaction, and restricted and repetitive patterns of behavior. ASD presents as a clinical spectrum, with variable levels of severity and multiple co-occurring conditions. The etiology of ASD may involve hundreds of genes and there is evidence that neurotransmitter and synaptic (NS) pathways are implicated. Proton Magnetic Resonance Spectroscopy (^1^H-MRS) has made it possible to study the concentration of brain neurometabolites and compare their levels in the brains of ASD and control individuals. We integrated genetic variants in NS genes with ^1^H-MRS analysis, and identified 12 predicted damaging variants (PDVs) in 12 NS genes in 10 ASD individuals, most mapping to genes involved in Gamma-aminobutyric acid (GABA) and glutamate pathways. Total creatine (tCr) and total N-acetyl aspartate (tNAA), markers of bioenergetics and neuronal metabolism, respectively, were lower in ASD patients with genetic alterations in NS genes compared to a control group without ASD. We conclude that PDVs in NS genes that are important for the regulation of glutamate or involved in GABAergic functions are associated with neurometabolic alterations, and that dysfunction in glutamatergic and/or GABAergic pathways may be implicated as these pathways are linked to the metabolic measures altered in cases.

**Supplementary Information:**

The online version contains supplementary material available at 10.1038/s41598-025-20090-x.

## Introduction

Autism Spectrum Disorder (ASD) is a neurodevelopmental condition characterized by social and communication deficits and repetitive behavioral patterns^1^. ASD is clinically and genetically very heterogeneous, presenting as a clinical spectrum of symptoms that are further complicated by multiple co-occurring conditions. The prevalent hypothesis proposes that a combination of genetic alterations with environmental exposure lead to ASD^2^. While hundreds of genes have been associated to ASD^3^, less than 40% of patients are diagnosed with genetic syndromes or have chromosomic alterations, single gene disorders, or gene variants and metabolic disturbances with mitochondrial dysfunction that can explain the disease etiology^4^.

Genes associated with ASD suggest the involvement of important physiological processes such as metabolism, chromatin remodeling, translation and synapse^5–8^. Experimental animal models of ASD provide evidence for compromised neurotransmission, synapse formation and stabilization, and synaptic plasticity mechanisms^9–12^. For instance, excitatory/inhibitory imbalance is often observed in ASD and thought to be due to abnormal glutamatergic excitatory and GABAergic inhibitory neurotransmission in several brain regions, affecting information processing and regulation of behavior^13^. Excitatory/inhibitory imbalance could potentially explain social and cognitive deficits associated with ASD^14,15^. Such imbalances may arise from alterations during the early stages of neural circuit development or involved in synaptic homeostasis, since several of the genes identified in linkage and association studies encode proteins involved in these processes^16,17^.

Several studies implicate synaptic genes encoding pre- and post-synaptic proteins in non-syndromic ASD, including synaptic adhesion molecules such as neuroligins, neurexins, cadherins and contactins^18–21^, synaptic scaffold proteins like the ProSAPs/SHANKs multidomain proteins^22^, ion channels like calcium channels^23^ and N-methyl-D-aspartate (NMDA) or Gamma-aminobutyric acid (GABA) neurotransmitter receptors^24,25^. Postmortem studies have discovered structural and functional alterations in glutamatergic and GABAergic pathways in ASD^26,27^. In addition, mutations in pre-synaptic genes such as *RIMS3* and post-synaptic genes such as *IL1RAPL1* and *SYNGAP1*, involved in either synaptic vesicle organization or synapse formation, were also associated with ASD^28,29^. While the biological validation of the involvement of the hundreds of genes that have been implicated in the disease is not trivial, because brain tissue is mostly available postmortem, a window to brain function and neurochemistry is offered by neuroimaging studies of in vivo metabolism. Conversely, identifying risk genes underlying structural, functional, and neurochemical alterations observed in the brain of ASD patients is important to provide a deeper understanding of the neurobiology of ASD. Proton Magnetic Resonance Spectroscopy (^1^H-MRS) has made it possible to study the cerebral concentration of key biochemical substances in clinical and non-clinical populations in a non-invasive way^30^. Specifically, ^1^H-MRS has revealed widespread neurochemical alterations in ASD, with a range of molecules and brain regions being implicated, but the picture is complex^31^. For instance, studies investigating glutamate and GABA have reported increased, decreased or unchanged levels of these metabolites in several brain regions^32^. However, these studies did not take individuals’ genetic background into account, which could help to further clarify how synaptic dysregulation contributes to ASD neurobiology. Given that prior studies have found changes in tNAA^15,33^, including a recent meta-analysis^34^ it is important to understand how this metabolite links to Glutamate and GABA, from a neurometabolic point of view. NAA is present almost exclusively in the central nervous system, and largely only in neurons. There is an intimate connection between glutamate and NAA, and other neurometabolites, based for example on precursor-product relationships^35^. Glutamate and aspartate levels can change in reverse ways. For example, a shift in the aspartate aminotransferase reaction leads to an increase in aspartate formation with a reduction of its glutamate precursor. Moreover, both glutamate and GABA metabolism are linked through intermediates of the tricarboxylic acid cycle, which explains changes in neurometabolic coupling with changes in the diet^36^ or in disorders such as diabetes^37^.

In spite of these methodological opportunities, the integration of genetic studies with brain imaging is still lagging behind, even though it can offer extremely important information to guide the development of novel therapeutic approaches^38^. In this study, we integrate genetic analysis and ^1^H-MRS to improve the understanding of how synaptic dysregulation contributes to ASD neurobiology. This approach may guide biomarker development for patient stratification, leading to more effective pharmacological therapies.

## Methods

### Sample population and clinical assessment of patients

In this study we analyzed 16 ASD cases, selected because we had comprehensive clinical information and genetic data from genomic scans as well as ^1^H-MRS information, and a control group of 14 subjects. The patients were recruited at the Autism Clinic from the Paediatric Hospital at the University Hospital in Coimbra, Portugal. All patients were previously diagnosed and assessed in the clinical setting at least twice a year by a team of neurodevelopmental pediatricians and psychologists with extensive experience in ASD assessment and diagnosis. Thus, a comprehensive clinical characterization was used to ensure that the patients met the inclusion and exclusion criteria by the time of the ^1^H-MRS session. Inclusion criteria for patients were: (i) age below 18 years old, (ii) full-scale intelligence quotient (FSIQ) above 70 as measured by the Portuguese versions of the Wechsler Intelligence Scales^39,40^, so to exclude subjects with intellectual disability, (iii) ASD diagnostic confirmed by positive results on the direct parental or caregiver interview performed by an experienced pediatrician, namely the Autism Diagnostic Interview - Revised (ADI-R)^41^, and proband direct assessment with Autism Diagnostic Observation Schedule (ADOS) performed by an experienced psychologist^42^.

Informed consent was obtained from the parents/guardians of the participants. Children and adolescents also gave oral informed consent.

The study was approved by the ethics committee of the Faculty of Medicine of the University of Coimbra and the Ethics Committee for Health from the Instituto Nacional de Saúde Doutor Ricardo Jorge, and was conducted in accordance with the declaration of Helsinki.

### Genetic data analysed

Genetic data included Copy Number Variants (CNVs) and Single Nucleotide Variants (SNVs), obtained in the context of two large ASD international consortia datasets: (1) the Autism Genome Project (AGP) used SNP arrays to carry out a Genome Wide Association Study (GWAS) with CNV detection, for 2611 families with one affected subject and the parents, from North America and Europe, including our team^43–45^; (2) the Autism Sequencing Consortium (ASC) performed whole exome sequencing (WES) for SNV detection in 10,008 individuals with ASD, and shared the output for research purposes^46^.

### Identification of predicted damaging variants in NS genes

We analyzed exomic data from the ASC. Quality control filters applied using the parameters defined in bcftools (https://samtools.github.io/bcftools/) were: Variant Quality Score VSQLOD= >-1.5; Read Depth DP > 8; Genotype Quality GQ > 20; Allelic Depth AD > 0.2 and Missingness < 10%. Predicted damaging SNVs in affected individuals were selected in genes from a well curated list of neurotransmission and synaptic candidate genes (NS genes) developed in a previous study^47^ and that resulted from the application of filters for these processes in different databases that combine information from several biological processes and the genes involved in those mechanisms, namely Gene Ontology (GO)^48^, KEGG pathway database^49^, Reactome^50^, SynaptomeDB^51^ and SynSysNet^52^. Variants with predicted damaging impact in protein function and/or structure were identified using the Ensemble Variant Effect Predictor (VEP) tool^53^. VEP determines the effect of a variant in genes, transcripts and protein sequences, as well as regulatory regions, and incorporates SIFT^54^ and PolyPhen-2 algorithms^55^ to score for changes to protein sequence. SIFT predicts whether an amino acid substitution affects protein function based on sequence homology and the physical properties of amino acids. PolyPhen-2 is a tool which predicts possible impact of an amino acid substitution on the structure and function of a human protein using physical and comparative considerations. We selected loss of function (LoF) variants (variants of frameshift, stop gain, start lost, splice acceptor and splice donor) and missense variants classified as deleterious by SIFT, and/or as possibly or probably damaging by Polyphen-2, according to the Ensembl VEP. We also selected variants classified as pathogenic or likely pathogenic by InterVar^56^. InterVar is a bioinformatics software tool for clinical interpretation of genetic variants by the American College of Medical Genetics and Genomics and the Association for Molecular Pathology (ACMG/AMP) 2015 guideline^57^. Finally, we filtered out the variants that were present in the large control datasets included in the Genome Aggregation Database (gnomAD)^58^; http://gnomad.broadinstitute.org/) with minor allele frequency (MAF) ≥ 5%. The gnomAD datasets were developed to provide large-scale data of genetic variation present in several populations, and can be used to filter out common benign/neutral variants and identify rare variants with clinical meaning based on the frequency in the human population. The dataset used (gnomAD v2.1.1) incorporates the information from 60146 controls sequenced as part of various disease-specific and population genetic studies. We further inspected the AGP dataset to identify NS genes targeted by CNVs in individuals with ASD. We only selected CNVs that were detected by more than one algorithm, as implemented in^44^.

### Characterization of predicted damaging variants in NS genes and associated biological pathways

We enriched in biological pathways the list of genes identified in the SNVs and CNVs analysis previously done, using the KEGG pathway database^49,59^ and the Reactome database^50^. GeneCards (https://www.genecards.org/) and PubMed (https://pubmed.ncbi.nlm.nih.gov/) were used to identify gene functions and the key roles of genes in the human brain. Gene expression in human brain regions was obtained from the Human Protein Atlas brain section (https://www.proteinatlas.org/humanproteome/brain). Additionally, we used the ClinVar database^60^ to inspect the classification of variants identified in this dataset based on supporting evidence, ClinVar classifies variants as pathogenic or benign, or as *V*ariants with *U*ncertain *S*ignificance (VUS) whenever a variant does not fulfill criteria for pathogenic or benign, or the evidence for benign and pathogenic is conflicting. It is important to take into consideration the difference between implicating a variant as pathogenic (causative) for a disease, and a variant that may be predicted to be damaging to the encoded protein, and not necessarily being implicated in a disease, since both classifications are independent^57^.

We also examined if the genes with the variants were identified in the SFARI list as ASD candidate genes, and the strength of the evidence associating these genes to ASD (https://gene.sfari.org/database/gene-scoring/). SFARI is an ASD dedicated database for the autism research community, containing up-to-date information on genes associated with ASD that incorporates a gene scoring module. This module establishes a gene rank according to the strength of the evidence that associates a given gene to the disease^61^. The strongest candidate genes (SFARI categories 1 and 2) come from well-defined evidence on human genetic studies. Category 1 considers rigorous statistical comparisons between cases and controls yielding genome-wide statistical significance with independent replication to be the strongest possible evidence for a gene, and in category 2 these criteria are slightly relaxed. Category 1 includes genes that have been clearly implicated in ASD, by the presence of at least 3 *de novo* likely-gene-disrupting mutations being reported in the literature, and are typically returned to the participants. Some of the genes in this category meet the most rigorous threshold of genome-wide significance (*p* < 5 × 10^− 8^); all at least meet a threshold of false discovery rate of < 0.1. Category 2 includes genes with 2 reported *de novo* likely-gene-disrupting mutations, and genes uniquely implicated by a genome-wide association study, either reaching genome-wide significance or, if not, consistently replicated and accompanied by evidence that the risk variant has a functional effect. Genes predisposing to ASD in the context of a syndromic disorder (e.g. Fragile X Syndrome) are placed in category (S) and, if there is additional evidence implicating them in ASD, will have a number in front of the S according to the strength of that evidence (1 and 2 are the strongest categories).

### Proton magnetic resonance spectroscopy (^1^H-MRS) data acquisition and analysis

^1^H-MRS acquisition, data processing and quality check were done in the context of a previous study^15^. ^1^H-MRS data was acquired in a 3T Siemens TimTrio scanner (Erlangen, Germany) using 12-channel birdcage head coil, without participant sedation following the procedure described in^15^. Briefly, a T1-weighted Magnetization Prepared Rapid Acquisition Gradient-Echo (MPRAGE) sequence was acquired for each participant with 1 × 1 × 1 mm3 voxel size, repetition time (TR) = 2.3 s, echo time (TE) = 2.98 ms, flip angle (FA) = 9°, field of view (FOV) = 256 × 256, 160 slices, and used for spectroscopy voxel positioning in the medial prefrontal cortex/anterior cingulate cortex. A single-voxel with 3 × 3 × 3 cm was acquired with the MEGA-PRESS method^62,63^. Acquisition parameters were: repetition time 1.5 s, echo time 68 ms, 392 averages (196 ON, 196 OFF), 1024 data points, bandwidth 2000 Hz with automatic shimming and chemical shift selective (CHESS) weak water suppression with a bandwidth of 35 Hz, resulting in an acquisition time of approximately 10 min. No saturation bands were used. LCModel version 6.3–1D (Stephen Provencher Inc., Oakville, Canada^15^ was used to quantify total N-acetyl aspartate (tNAA) (n-acetylaspartate + n-acetylaspartilglutamate), gamma aminobutyric acid + macromolecules (GABA+) and glutamate + glutamine (Glx) from the ON-OFF difference spectrum, and total creatine from the OFF spectrum, which were corrected for the amount of cerebrospinal fluid (CSF-corrected).

Metabolites were quantified in three groups: (1) controls without ASD (*n* = 14); (2) subjects with ASD without predicted damaging variants (PDVs) in NS genes (*n* = 6); (3) subjects with ASD with PDVs in NS genes (*n* = 10). Statistical analysis of spectroscopy data was done with IBM SPSS statistics version 23 (IBM Corporation, IL, USA). Data distributions were assessed for normality (Shapiro–Wilk test). Normally distributed variables were analyzed using one-way ANOVA (Tukey correction for multiple comparisons was applied), while non-normally distributed variables were analyzed with the Kruskal-Wallis test, with metabolite (or ratio) as dependent variable and group as independent variable to test for differences across the 3 groups. An additional trend analysis was performed using the Jonckheere-Terpstra. Significance level was *p* = 0.05.

A post hoc power analysis was carried out with G*Power 3.1.9 software^64,65^ to evaluate the statistical power of the significant between groups results.

## Results

### Population characterization

In the present study, we analysed 16 patients and 14 control participants. The three groups defined were matched for age and performance intelligence quotient (Table 1). The two ASD groups were matched for full-scale intelligence and verbal quotients, but were different from the control group (post hoc Tukey *p* < 0.002) (Table 1).

**Table 1:** Demographic, neuropsychological and neurodevelopmental information.

	Control (*n* = 14)		No (*n* = 6)		Yes (*n* = 10)	
	Mean ± SD	Range	Mean ± SD	Range	Mean ± SD	Range
Age	13 ± 2	10–18	14 ± 2	13–18	13 ± 2	11–17
FSIQ	121 ± 20	87–154	90 ± 7	79–100	94 ± 13	70–112
VIQ	123 ± 22	84–155	89 ± 17	66–117	95 ± 10	72–112
PIQ	111 ± 17	95–146	96 ± 20	64–124	97 ± 15	73–117
ADI-R Social interaction			13 ± 5	11–23	18 ± 5	11–25
Communication			12 ± 5	7–20	11 ± 2	8–14
Repetitve behaviors			4 ± 1	3–5	6 ± 2	3–8
Developmental delay			3 ± 2	0–5	3 ± 2	0–5
ADOS Global Result			14 ± 3	12–19	15 ± 4	10–19
Communication			5 ± 1	5–6	5 ± 1	3–7
Social interaction			8 ± 3	6–13	10 ± 3	7–14

Abbreviations: Control, participants without ASD; No, ASD individuals without predicted damaging variants in NS genes; Yes, ASD individuals with predicted damaging variants in NS genes; n, number of participants; SD, standard deviation; FSIQ, full-scale intelligence quotient; VIQ, verbal intelligence quotient; PIQ, performance intelligence quotient; ADI-R, Autism Diagnostic Interview – Revised; ADOS, Autism Diagnostic Observation Schedule.

### Identification and characterization of predicted damaging variants in NS genes in the cases analysed

We identified 12 PDVs in NS genes in 10 of the cases analyzed, namely 10 SNVs and 2 CNVs (Table 2, and Supplementary Table 1 for a complete description of variants). Two patients had 2 variants in different genes, while all others had only 1 (Supplementary Table 1). Table 2 shows a summary of the characterization of variants. Most of the variants identified in the cases analysed are in genes involved in GABA and glutamate pathways. Additionally, 1 variant was found in the gene *CHRND* involved in the Acetylcholine pathway. The majority of the SNVs are classified by VEP as missense variants, except one stop-gain variant, and the two CNVs are deletions (Table 2 and Supplementary Table 1). Additionally, we checked if any of these variants are reported in the ClinVar database to inspect the classification of variants based on additional supporting evidence. ClinVar classifies variants as pathogenic or benign, or VUS whenever a variant does not fulfill criteria for pathogenic or benign or the evidence for benign and pathogenic is conflicting. Of 6 variants reported in the ClinVar database, 5 are VUS and 1 is classified as pathogenic. This pathogenic variant is found in the gene *ALDH5A1* that encodes an enzyme that catalyzes the dehydrogenation of gamma-aminobutyraldehyde to GABA, and is related with Succinic semialdehyde dehydrogenase deficiency, which is characterized by infantile-onset hypotonia, developmental delay, cognitive impairment, expressive language deficit, and mild ataxia (Table 2 and Supplementary Table 1).

We also analyzed whether NS genes affected by PDVs in these 10 cases are part of the SFARI gene list of ASD candidates. Of 5 genes with PDVs involved in GABA metabolism (*VPS18*,* ALDH5A1*,* GAD1*,* ALDH9A1*,* SYNE1*), 1 has a SFARI score of 1 S (*ALDH5A1*) and another has a score of 2 (*SYNE1)*, and of 6 genes with PDVs involved in the glutamate pathway (*CREBBP*,* AKAP9*,* EPB41L2*,* RTN3*,* CACNA1C*,* PANX1*), 2 have a SFARI score of 1 S (*CREBBP* and *CACNA1C*) and another has a score of 2 (*AKAP9*) (Table 2 and Supplementary Table 1). Categories 1 and 2 are the strongest SFARI categories with well-defined evidence on human genetic studies. An “*S*” is added to the numeric score when the gene also predisposes to ASD in the context of a syndromic disorder.

**Table 2:** Summary of the characterization of predicted damaging variants (PDVs) in NS genes in 10 ASD cases.

NS pathway involved	Cases (*N*)	Gene symbol	Variant type	Position	Variant characterization	ClinVar report	SFARI gene score
GABA	5	*VPS18*	SNV	15-41193187-A-G	Missensse, Heterozygotic	Not reported in ClinVar	-
		*ALDH5A1*	SNV	6-24528285-C-T	Stop_gain, Heterozygotic	Pathogenic​	1 S
		*GAD1*	SNV	2-171713633-G-A	Missensse, Heterozygotic	VUS​	-
		*ALDH9A1*	SNV	1-165664551-A-G	Missensse Heterozygotic	VUS​	-
		*SYNE1*	SNV	6-152532698-C-T	Missensse, Heterozygotic	VUS​	2
Glutamate	6	*CREBBP*	SNV	16-3843551-C-T	Missensse, Heterozygotic	Not reported in ClinVar	1 S
		*AKAP9*	SNV	7-91630565-T-C	Missensse, Heterozygotic	VUS​	2
		*EPB41L2*	CNV	6-131365548-131395373	Deletion encompassing *EPB41L2* gene	Not reported in ClinVar	-
		*RTN3*	SNV	11-63487475-G-C	Missensse, Heterozygotic	Not reported in ClinVar	-
		*CACNA1C*	CNV	12-2115897- 2127756	Deletion encompassing *CACNA1C* gene	Not reported in ClinVar	1 S
		*PANX1*	SNV	11-93886666-T-C	Missensse, Heterozygotic	Not reported in ClinVar	-
Acetylcholine	1	*CHRND*	SNV	2-233396064-G-A	Missensse, Heterozygotic	VUS	-

NS: neurotransmitter and synaptic; GABA: gamma aminobutyric acid; VUS: Variants with Uncertain Significance.

### Magnetic resonance spectroscopy

**Fig. 1:**
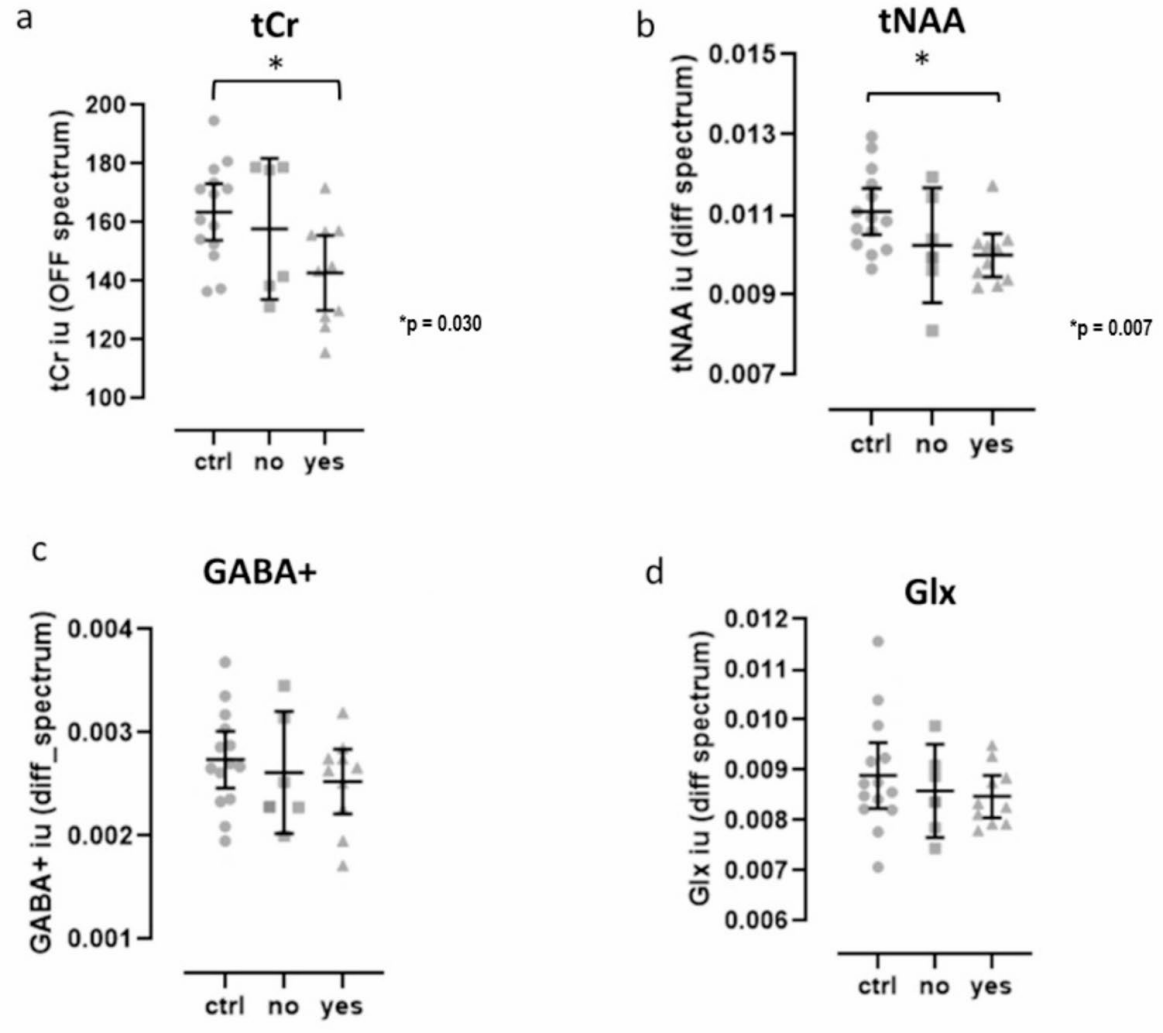
Metabolites quantified in the medial prefrontal cortex/anterior cingulate cortex of ASD individuals and controls without ASD. Circles: controls without ASD (ctrl, *n* = 14); Squares: ASD individuals without predicted damaging variants in NS genes (no, *n* = 6); Triangles: ASD individuals with predicted damaging variants in NS genes (yes, *n* = 10); (**a**) tCr: total creatine (creatine + phosphocreatine); (**b**) tNAA: total N-acetyl aspartate (n-acetylaspartate + n-acetylaspartilglutamate); (**c**) GABA+: gamma aminobutyric acid + macromolecules; (**d**) Glx: glutamate + glutamine. Graphs depict individual values, mean ± 95% confidence interval. * P Significance of the trend analysis using the the Jonckheere-Terpstra test.

One-way ANOVA revealed significant differences for tNAA levels (F_(2,27)_ = 3.734, *p* = 0.037, η² = 0.217, 95% CI [0.000, 0.420] ). Post hoc Tukey test for multiple comparisons showed significant differences between the control group without ASD and the ASD group with genetic alterations in NS genes, such that tNAA levels were lower in the ASD group with such genetic alterations (*p* = 0.038, 95% CI: [0.002, 0.00005], Cohen’s d = 0.114). The post hoc power analysis based on the tNAA effect size indicated a power of 0.68 at α = 0.05. The tCr levels showed a near threshold level difference (Kruskal-Wallis: H(2) = 5.583, *p* = 0.061). A trend pattern of decreased levels for all metabolites across the 3 groups was observed (Fig. 1), such that metabolite levels decreased from control group > ASD without genetic alterations > ASD with genetic alterations in the ASD group with PDVs identified, which was significant for tCr (J = 86.00, *p* = 0.030) and tNAA levels (J = 73.00, *p* = 0.007) (Fig. 1). The effect size of this significant trend was calculated as Z/√N (rank bi-serial correlation), and was *r* = 0.40 (95% CI [0.05, 0.66]) and *r* = 0.49 (95% CI [0.16, 0.72]), respectively, which is considered a moderate effect size. There were no statistical differences between groups or significant trends for GABA + and Glx analysis.

##  Discussion

 In this study, we investigated how synaptic dysregulation contributes to ASD integrating measures of brain metabolites through ^1^H-MRS analysis with data on genetic variation. In line with the trend analysis, the levels of tCr and tNAA were significantly lower in the ASD group with PDVs in NS genes when compared with the control group. While the post hoc power analysis based on the tNAA effect size already indicated a power of 0.68 at α = 0.05, this actually underestimates the power of the performed trend analysis, which detected a significant monotonic trend of moderate effect size using the Jonckheere-Terpstra test. This method is more powerful than a one-way ANOVA when evaluating ordered differences, even with non-parametric data, and accordingly also revealed a significant trend for the tCr levels, further supporting the main conclusions of the study. Nonetheless, future research with larger cohorts and independent replication, potentially through multicenter collaborations, will be useful to validate and extend the current results.

The results are in agreement with the notion that tNAA is one of the most consistently altered neurometabolites in ASD as we observed before^15^, and was confirmed recently^33,34^. Its metabolism is tightly linked to the regulation of Glutamate and GABA levels. We further observed that the NS genes with PDVs in 10 of the individuals analysed encode proteins implicated either in GABA or glutamate functions.

The genes *VPS18*,* SYNE1*,* ALDH9A1*,* ALDH5A1* and *GAD1* are implicated in GABA pathways. The gene *VPS18* is involved in dendrite development of Purkinje cells, which are a class of GABAergic inhibitory neurons located in the cerebellum^66^. The *SYNE1* gene is also expressed in the Purkinje cells and is important for movement coordination^67^. The gene *ALDH9A1* encodes an enzyme that catalyzes the dehydrogenation of gamma-aminobutyraldehyde to GABA^68,69^. The gene *ALDH5A1* also encodes an aldehyde dehydrogenase family member that catalyzes one step in the degradation of GABA. Diseases associated with *ALDH5A1* include Succinic Semialdehyde Dehydrogenase Deficiency (SSADHD), which is an autosomal-recessively inherited disorder characterized by global developmental delay, hypotonia or Epilepsy^70^. Interestingly, guanidino-species involved in the production of creatine are altered in SSADHD^71^ which may provide clues for the participation of *ALDH5A1* in some of the alterations in tCr levels observed. The *GAD1* gene is responsible for encoding an isoform of the enzyme glutamic acid decarboxylase (GAD1) that converts glutamate to GABA^72^.

The genes *PANX1*,* CACNA1C*,* RTN3*,* EPB41L2*,* AKAP9* and *CREBBP* are involved in glutamatergic function. The *PANX1* gene encodes a protein that forms plasma membrane channels that play a role in the release of ATP and glutamate in neurons and astrocytes^73,74^. The gene *CACNA1C* is involved in glutamate release and in the regulation of calcium concentration in the intracellular space^75^. Similarly, studies show that the *RTN3* gene has been implicated in early phases of the glutamate secretory pathway^76,77^. The protein encoded by the gene *EPB41L2* interacts with other proteins to regulate the insertion of AMPA receptors (AMPARs) which play a critical role in glutamatergic neurotransmission. The gene *AKAP9* is also involved in glutamatergic signaling and is part of a protein network implicated in ASD^78,79^. The gene *CREBBP* also participates in the modulation of the glutamatergic neurotransmission as is involved in long-term potentiation mechanisms in glutamatergic synapses^80^. Most variants indentified in this study don´t have a clear outcome. Functional validation is needed to understand their impact in protein function to consolidate our results and, eventually, identify drug targets.

Our results are in accordance with other studies that reported reduced levels of NAA, glutamate, glutamine and GABA in children with ASD^31,34^. ^1^H-MRS studies measuring metabolite concentrations in participants with ASD have shown inconsistent findings, particularly for glutamate, GABA, NAA, and creatine; however, some patterns are evident. Lower GABA and NAA concentrations are frequently observed, suggesting that altered brain metabolism, particularly regarding neuronal integrity and excitation/inhibition balance, may be implicated in the pathophysiology of ASD^34^. Creatine levels can fluctuate with age and brain region. Some studies have found higher Cr levels in adults with ASD compared to children, with specific increases in the temporal lobe and decreases in the occipital lobe^31^. Age-related differences in metabolite concentrations are observed in ASD, with some metabolites showing distinct patterns in children and adults. The inconsistency of findings can also be attributed to differences in the MRS methodology used (e.g., voxel size, echo time, repetition time), as well as variations in sample demographics (e.g., age, sex, severity of ASD symptoms)^31^. Different methods for data analysis, including metabolite ratios calculations and data normalization, can also contribute to the variability on results. Understanding the specific metabolite alterations in ASD may help developing targeted therapies that address the underlying neurobiological mechanisms.

Multiple lines of evidence indicate that the dysfunction of glutamate receptors such as NMDARs is associated with ASD^81,82^. NMDAR is an ionotropic glutamate receptor (iGluR) that is activated by the neurotransmitter glutamate^83^ and mediates excitatory synaptic neurotransmission, which is important for synaptic plasticity, learning and memory. Earlier studies demonstrated that NMDARs are fundamental during neuronal development and synapse maturation processes^84^. Pharmacological enhancement or suppression of NMDAR function attenuate social withdrawal and repetitive behaviors^81^. On the other hand, GABA is an important inhibitory neurotransmitter in the central nervous system and there is evidence for an association between mutations affecting GABA function and ASD, including studies that reported reduced expression of GABAergic genes and GABA-related proteins in postmortem brain samples from individuals with ASD^85^. One of the most commonly reported chromosomal loci where CNVs observed in ASD in the 15q11-q13 chromosomal region^86,87^ that contains a number of genes encoding subunits of GABA receptors. GABA-A and GABA-B receptors alterations were identified in ASD brains^88–91^. Additionally, postmortem studies and experiments with animal models support GABAergic dysfunction playing a role in ASD^85,92^. It is estimated that Epilepsy co-occurs in 30% of ASD patients, probably in consequence of an excitation and inhibition imbalance. There are studies that estimated that subclinical epileptiform activity may occur in up to 85% of children with ASD^93,94^. Altogether, several lines of evidence support the imbalance of excitation to inhibition ratio proposal in ASD, possibly caused by alterations in GABA and glutamate pathways^95^.

The present study shows that having PDVs in NS genes might have a marked impact in brain neurochemistry. Specifically, we observed significant alterations in tCr (bioenergetics) and tNAA (neuronal metabolism and neuromodulation) levels when compared to the levels measured in the control group without ASD. We showed that almost all the variants identified involved glutamate and GABA pathways and there is previous evidence of the interaction between NMDARs and GABA with N-acetylaspartate (NAA) and creatine (Cr). Creatine modulates GABAergic and glutamatergic pathways^96^, and creatine treatment was shown to promote the differentiation of GABAergic neuronal precursors in cultured fetal rat spinal cord^97^ and GABAergic cells in cultured striatal tissue^98^. In addition, previous works suggest that creatine may also modulate brain NMDARs^99,100^ and exert positive cognitive effects through this action^101^. Other compounds may also act on brain NMDARs on oligodendrocyte function, and may interfere in the myelination of axons. Demyelination is associated to several disorders such as leucodystrophies and there is evidence that this can reflect an action of NAA on oligodendrocyte NMDA receptors^102^. The myelination of axons by oligodendrocytes is essential for normal brain activity^103^, and failures in this process results in compromised mental and physical activity. The activation of glutamate receptors on oligodendrocytes or their precursor’s cells can contribute to the loss of myelin^102^. Glutamate release was shown to kill precursor and immature oligodendrocytes^104,105^, and the activation of oligodendrocytes NMDARs by glutamate causes demyelination and loss of axonal action potential propagation^106,107^. However, the role of NMDA receptors in oligodendrocytes has been controversial. Previous studies showed that transient NMDA-receptor activation induces a Ca^2+^ -dependent efflux of NAA in rat hippocampus. There is evidence suggesting that Ca^2+^ -influx via the NMDAR modulates the efflux of NAA from neurons and may contribute to pathologies^108^. More recently, it was shown that NMDARs also have a role in glucose uptake regulation in response to axonal glutamate release, modulating the cooperation between oligodendrocytes and axons^109^. A role for NMDA receptor signaling in oligodendrocyte differentiation and promoting myelination has been suggested^25,110^. Increasing evidence suggests that myelination is a dynamic process that may be disrupted in the ASD brain^111^. NAA catabolism in oligodendrocytes is a precursor to fatty acids involved in the myelination of neuronal axons^112^. A pilot study in children with ASD suggested that alterations in axons and myelin of the corona radiata appear to be associated with the clinical severity of ASD^111,113^. Importantly, the ^1^H-MRS tNAA signal is a combination of NAA and n-acetylaspartilglutamate (NAAG) which may contribute to 10–25% of the total signal captured^114^. NAAG is thought to play a role in neuroprotection and synaptic plasticity due to modulatory properties of neurotransmission involving glutamate, GABA and other monoamines^115^. Thus, while no Glx or GABA + differences were directly observed, these neurotransmitters are tightly linked to brain bioenergetics and metabolism measures that were altered, as explained above. Importantly, ^1^H-MRS captures the bulk levels of metabolites and is not able to discriminate between the metabolic and neurotransmitter pools which are in constant flow^112^. Hence, we cannot exclude that synaptic glutamate and GABA levels are altered in the ASD participants with genetic alterations. Future studies disentangling the neurotransmitter and neurometabolic pools, for example using carbon-labeled spectroscopic approaches^116^, could provide further insight.

The interconnection between the observed metabolite alterations and ASD symptoms is still under investigation. NAA is a marker of neuronal integrity and its levels can be affected by several factors, including mitochondrial dysfunction^117^. Instead of directly aiming to increase NAA levels, research have focused on addressing underlying neurological and metabolic factors that may contribute to ASD symptoms and potentially improve overall neuronal health^118,119^. Potential treatments and interventions may include nutritional approaches that may help improve mitochondrial function, which is implicated in some cases of ASD. Dietary guanidinoacetic acid (GAA) has been shown to increase brain creatine levels. Studies have demonstrated that supplementing with GAA can lead to a notable increase in creatine levels in several brain regions, which may improve energy production and utilization within the brain^120^. Experiments with Dodecyl creatine ester (DCE), a prodrug of creatine, are very promising in increasing brain creatine concentrations and improving cognitive function in mice^121^.

 In conclusion, the present study shows that the ASD cases analysed which have significant alterations in tNAA and tCr levels also have PDVs in genes that are important for processes associated with the regulation of glutamate and GABAergic functions. According with evidence resulting from previous studies, it is possible that the implications of the ^1^H-MRS alterations detected in the patients of the present study are associated with glutamatergic and/or GABAergic pathways dysregulation.

## Supplementary Information

Below is the link to the electronic supplementary material.

Supplementary Material 1

## Data Availability

Sequence data that support the findings of this study was downloaded from the database of Genotypes and Phenotypes (dbGaP) web site, under dbGaP accession phs000298.v1.p1; phs000298.v2.p2; phs000298.v3.p2; phs000298.v4.p3. Version 1–3: The data sets were deposited by the ARRA Autism Sequencing Collaborative, an ARRA-funded research initiative. The datasets generated and/or analysed during the current study are available from the corresponding author on reasonable request.
